# Identification of putative *cis*-regulatory elements in *Cryptosporidium parvum *by *de novo *pattern finding

**DOI:** 10.1186/1471-2164-8-13

**Published:** 2007-01-09

**Authors:** Nandita Mullapudi, Cheryl A Lancto, Mitchell S Abrahamsen, Jessica C Kissinger

**Affiliations:** 1 Department of Genetics & Center for Tropical and Emerging Global Diseases, Paul D. Coverdell Center, D.W. Brooks Dr., University of Georgia, Athens, GA 30602, USA; 2 Veterinary and Biomedical Sciences, University of Minnesota, St Paul, MN 55108, USA

## Abstract

**Background:**

*Cryptosporidium parvum *is a unicellular eukaryote in the phylum Apicomplexa. It is an obligate intracellular parasite that causes diarrhea and is a significant AIDS-related pathogen. *Cryptosporidium parvum *is not amenable to long-term laboratory cultivation or classical molecular genetic analysis. The parasite exhibits a complex life cycle, a broad host range, and fundamental mechanisms of gene regulation remain unknown. We have used data from the recently sequenced genome of this organism to uncover clues about gene regulation in *C. parvum*. We have applied two pattern finding algorithms MEME and AlignACE to identify conserved, over-represented motifs in the 5' upstream regions of genes in *C. parvum*. To support our findings, we have established comparative real-time -PCR expression profiles for the groups of genes examined computationally.

**Results:**

We find that groups of genes that share a function or belong to a common pathway share upstream motifs. Different motifs are conserved upstream of different groups of genes. Comparative real-time PCR studies show co-expression of genes within each group (in sub-sets) during the life cycle of the parasite, suggesting co-regulation of these genes may be driven by the use of conserved upstream motifs.

**Conclusion:**

This is one of the first attempts to characterize *cis*-regulatory elements in the absence of any previously characterized elements and with very limited expression data (seven genes only). Using *de novo *pattern finding algorithms, we have identified specific DNA motifs that are conserved upstream of genes belonging to the same metabolic pathway or gene family. We have demonstrated the co-expression of these genes (often in subsets) using comparative real-time-PCR experiments thus establishing evidence for these conserved motifs as putative *cis*-regulatory elements. Given the lack of prior information concerning expression patterns and organization of promoters in *C. parvum *we present one of the first investigations of gene regulation in this important human pathogen.

## Background

*Cryptosporidium parvum *is an apicomplexan parasite that causes diarrhea in humans and livestock and is recognized as a common opportunistic and potentially life-threatening pathogen in AIDS patients. It is therefore considered a major public health problem [1]. *Cryptosporidium parvum *has a complex, obligate intracellular life cycle that is characterized by a series of asexual and sexual developmental stages. Infection is initiated by the ingestion of environmentally resistant oocysts that release sporozoites capable of invading intestinal epithelial cells. The obligate intracellular nature and complex life cycle make it difficult to study the developmental biology of the organism. Purification of the parasites from host cells is currently impossible. The different life cycle stages cannot be reproduced under *in-vitro *conditions [2,3]. The situation is complicated further by the fact that the parasite is not amenable to either long-term cultivation or genetic dissection. Clearly, alternative approaches to investigate fundamental gene regulatory mechanisms in this important pathogen are required. Analysis of genomic sequence data and RT-PCR are two of the few available options. Genomes of two *Cryptosporidium *species (*C. parvum *and *C. hominis*) have recently been sequenced [4,5]. The animal pathogen *C. parvum *has a highly A+T rich (70%), compact genome of 9.1 Mb comprising 8 chromosomes that are believed to encode 3952 protein coding genes separated by very short intergenic spaces of around 0.5 kb. Only 5–20% of the genes are thought to contain introns. Sequence analysis has revealed a reduced transcriptional and regulatory apparatus in comparison to other eukaryotes [4,5].

**Figure 1:**
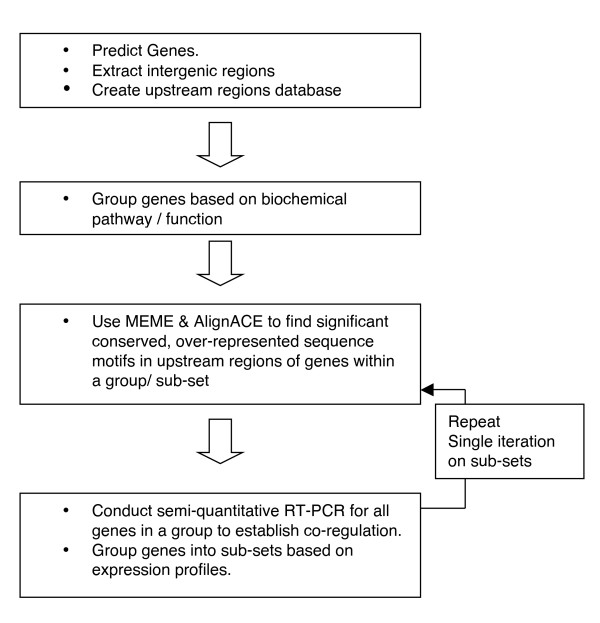
Flow-chart illustrating methodology. Pattern-finding was carried out in two iterations, first *de novo *and a second time using information obtained from comparative real-time PCR expression profiles.

The study of promoters and *cis*-regulatory elements in apicomplexan parasites presents an interesting challenge. A few gene-specific experiments in the apicomplexan parasite *Toxoplasma gondii *have revealed the absence of canonical elements such a TATA box in promoter regions. Instead, independent gene-specific studies have identified other motifs to be significant in gene-expression [6-8]. In *C. parvum*, experimental analysis of promoters and gene expression, including microarrays, is currently not possible due to the aforementioned experimental limitations. However, the availability of two complete genome sequences and several bioinformatics tools to mine sequence data offer alternative approaches to identifying putative cis-acting promoter elements. We undertook a computational approach to identify conserved, over-represented DNA motifs in the intergenic regions of the genome that could serve as putative *cis-*regulatory elements. We have made an attempt to characterize regulatory elements in the absence of any known elements and limited expression data for a select number of genes. Following data mining analyses, we correlated our computational findings with independent experimental analyses (Figure 1). Our strategy involved grouping genes based on function and mining for conserved motifs in the upstream intergenic regions. We applied two pattern finding algorithms MEME [9] and AlignACE [10] to identify conserved, over-represented DNA motifs. We then employed comparative real-time PCR to establish the expression profiles of the genes examined.

## Results

### Genes with conserved upstream motifs have similar expression profiles

The groups of genes selected in this study are involved in parasite-specific functions as well as housekeeping-type activities (Table 1). Parasite-specific gene families included genes encoding cryptosporidial oocyst wall proteins (COWPs) and large secretory proteins (Cp LSPs), genes known to show concerted post-infection expression patterns [4,11]. The housekeeping genes used in the analysis included genes involved in nucleotide salvage, DNA replication and glycolysis. We show that each of these groups of genes share different conserved upstream motifs. No common, general motif was conserved across all groups, barring AT-rich stretches, which were not statistically significant given the AT-richness of the genome.

Algorithms for pattern finding can report several hundred motifs ordered by their statistical significance as compared to a background model. Since statistical significance alone is not a sufficient indicator of biological significance, we applied a rule-based approach to identify candidate motifs that warrant further investigation. Candidate motifs were required to be within the top ten motifs predicted by both algorithms; display high information content and preferably show multiple occurrences within each upstream region. Information content as determined by MEME depends on the frequencies of the bases in a given column compared to the overall frequencies of those bases in the group of sequences. The more conserved the position is and the more rare the conserved nucleotides are, the higher the information content is. We provided MEME with a background file containing all inter-coding regions in the genome against which information content was calculated for each motif. (See additional files 2 and 3 for the scores, E-values and information content of reported motifs).

Each group of genes that shared conserved, upstream motifs was examined for correlated expression profiles via comparative real-time PCR at 6 different post-infection time points. The housekeeping groups of genes were further resolved into three sub-sets based on their expression profiles. For each sub-set, a second iteration of pattern finding was performed to determine if conserved motifs within each sub-set existed. We find a correlation between genes that contain distinct, conserved, upstream motifs and their corresponding expression profiles over a 72 h post-infection period.

### Genes encoding the COWP family

**Figure 2:**
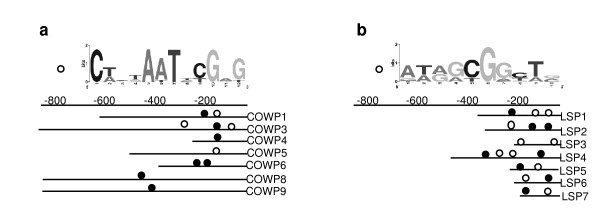
Motifs identified upstream of oocyst wall and large secretory proteins. (a) Upstream regions of genes encoding cryptosporidial oocyst wall proteins, and the occurrences of the most significant upstream motif shared by all of these upstream regions. The positions of the motifs are drawn to scale. All positions are shown with respect to the translational start. Solid black symbols denote a motif located on the reverse strand. Sequence-logo displaying the information content for the over-represented motif. (b) Upstream regions of genes encoding cryptosporidial large secretory proteins, and the occurrences of the most significant upstream motif shared by all of these upstream regions. Sequence-logo displaying the information content for the over-represented motif. Expression profiles for both families of genes were published elsewhere (Abrahamsen *et al*. 2004; Templeton *et al*. 2004).

The cryptosporidial oocyst wall proteins comprise a multi-gene family that demonstrate a defined pattern of expression during *in vitro *development, with expression levels peaking at 48 h through 72 h post-infection [11]. Genes encoding members of this family are scattered throughout the genome and not clustered in a tandem array. The most significant motif found in the upstream regions of these genes as determined by both algorithms was a 12 bp motif (Figure 2a). This motif is present one or two times in the upstream region of all COWP genes, and when present in pairs, the motifs are often within 50 – 100 bp of each other. The promoter regions of this gene family are not alignable outside of the conserved motifs identified, indicating that the conserved motif is not simply a function of recent gene & promoter duplication.

### Genes encoding the Cp LSP family

The large secretory proteins comprise a gene family that shows genomic co-localization in a cluster on chromosome 7. They are also co-expressed during the life cycle [4]. Figure 2b shows a single DNA motif found upstream of each of these genes, with a well-conserved sequence. This motif occurs 2–3 times in all of the upstream regions, and is often located within -350 bp from the translational start. As is the case with the COWP gene family, the promoter regions of this gene family are also not alignable outside of the conserved motifs identified, indicating that the conserved motif is not simply a function of recent gene & promoter duplication.

### Genes involved in nucleotide metabolism

**Figure 3:**
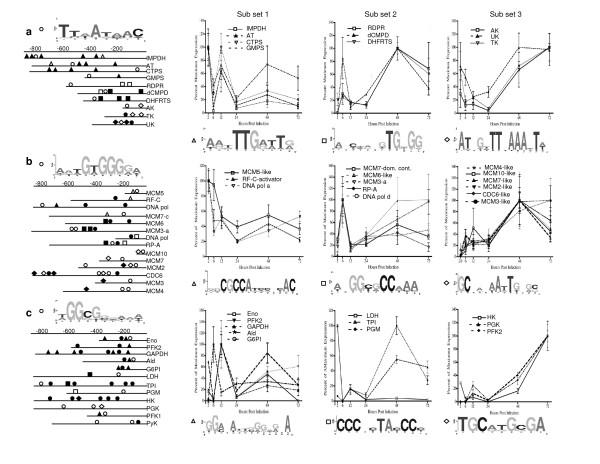
Results of motif and expression analyses. (a) Motifs and expression profiles associated with genes involved in nucleotide metabolism. Schematic representation of the upstream regions are shown for each gene. The location of 4 different candidate motifs are indicated by the use of four different shapes. The single motif found in each gene of the group is indicated by a circle. The locations of three additional candidate motifs, each associated with a sub-set of sequences are indicated by the remaining shapes drawn on the upstream regions and as indicated to the left of each sequence logo. Solid black shapes indicate motifs found on the reverse strand. Comparative real-time PCR profiles for sub-sets of each group of genes organized by expression profile over a 72 h period are shown as sub-sets 1–3 Each sub-set is associated with a single candidate motif. (b) Motifs and expression profiles associated with genes involved in DNA replication. (c) Motifs and expression profiles associated with genes involved in glycolyis.

*Cryptosporidium parvum *possesses highly streamlined nucleotide metabolic pathways, relying on the host cell for the salvage of both purine and pyrimidine residues. These pathways also contain genes that have been transferred into the nuclear genome of *C. parvum *from bacteria and plants via intracellular or horizontal gene transfer. The essential functions of these genes and their distinct evolutionary origin make them important drug-targets in developing anti-cryptosporidial chemotherapy [12]. We examined ten genes involved in nucleotide salvage and modification to identify significant motifs common to their upstream regions. We could not find a significant motif reported by both algorithms to be present upstream of all of the genes. MEME alone reported an 8 bp AT-rich motif present at least once in all the sequences at varying positions from the translational start (Figure 3a). Based on their real-time PCR expression profiles, the genes were divided into 3 sub-sets (sub-set 1, 2 and 3). Sub-set 1 contained three enzymes involved in the transport and modification of purines (AT, IMPDH and GMPS), and also one pyrimidine-modifying enzyme (CTPS). These genes were characterized by high expression levels at 2 h and 12 h post-infection and the most significant motif specific to this sub-set was a 10 bp motif shown (Figure 3a, sub-set 1). This motif was often found multiple times in the upstream regions and almost always found on the reverse strand. Three remaining pyrimidine-modifying enzymes (RDPR, dCMPD and DHFR-TS) had expression levels that peaked at 48 h post-infection and dropped subsequently. They comprise sub-set 2. These genes contained a 12 bp motif in their upstream regions, also seen at varying positions from the translational start (Figure 3a, sub-set 2). The three kinases (AK, TK and UK) involved in nucleotide salvage were grouped together in sub-set 3 based on their high expression levels at 48 h and 72 h post-infection. They were found to contain a conserved 14-bp AT-rich motif in their upstream regions (Figure 3a, sub-set 3).

### Genes involved in DNA replication

Analysis of the *C. parvum *genome reveals that the organism possesses a reduced complement of genes involved in DNA replication [4]. We chose to study genes involved in DNA replication expecting that they would be co-regulated in a time-dependent manner associated with the life cycle [13,14]. The most significant motif identified by both MEME and AlignACE was a single G-rich motif present upstream of all of the genes occurring multiple times in some of the upstream regions (Figure 3b). These genes could be resolved into 3 sub-sets based on their comparative RT-PCR expression profiles. Three genes peaking at 2 h post-infection were classified into sub-set 1. A 14 bp motif with a core conserved 5'-CGCCAA-3' sequence was found occurring once upstream of these three genes (Figure 3b, sub-set 1). At 6 h post-infection, a few genes coding for MCM-like proteins and the single-stranded binding protein RP-A were found to peak in expression levels. These were classified into sub-set 2. The most significant motif found specific to this sub-set was an 11 bp motif occurring one or two times in the upstream regions of this sub-set (Figure 3b, sub-set 2). Most of the MCM-like proteins peaked at 48 h post-infection and were classified into sub-set 3. These genes were found to contain a 13 bp motif, with a relatively less-conserved sequence, present one or more times in their upstream regions (Figure 3b, sub-set 3).

### Genes involved in glycolysis

Glycolysis is considered to be the main source of energy in the Coccidia, and especially so in *C. parvum *due to the lack of evidence of a mitochondrion and a functional respiratory chain [4,15,16]. Ten genes associated with glycolysis were considered for this study, and a single motif was found to be over-represented in all of their upstream regions (Figure 3c). This 10 bp motif contains a core 5'-GGCG-3' sequence and is present multiple times in some of the upstream regions. No outstanding pattern with respect to the orientation or position relative to the start of translation is apparent. Comparative real-time PCR experiments resolved the glycolytic genes into three sub-sets based on their expression profiles during development. The genes that peaked at 6 h- 12 h post-infection were included in sub-set 1. The most significant motif found upstream of these genes was a 14 bp motif occurring uniquely, or many times and almost always on the opposite strand (Figure 3c, sub-set 1). Sub-set 2 is comprised of 3 genes exhibiting weakly correlated expression profiles, two of which peak at 48 h post-infection. An 11 bp C-rich motif was found to occur one time in each of their upstream regions (Figure 3c, sub-set 2). We are encouraged that a conserved motif was found for this group, however, we remain unconvinced about the validity of this group based on their expression profiles alone since there is a discrepancy in the profiles. Sub-set 3 consists of 3 genes with expression levels peaking at 72 h post-infection. A 9 bp motif was found to be common to their upstream regions. This motif was very well conserved at the sequence level (TGC [A/G] [T/G]G [C/G]GA) (Figure 3c, sub-set 3) and was found occurring once upstream each gene.

### Genome-wide occurrences of candidate motifs

The candidate motifs reported in this study were selected based on rules as described earlier. In all the cases except one, these motifs are also found elsewhere in the genome, upstream of other genes. This is not surprising given that these are short sequences, with several degenerate positions. Expression profiles are not yet available for every gene to test if the other genes containing these motifs upstream also display similar expression profiles. (See additional file 3).

### Comparative studies in *C. hominis *and other apicomplexans

For each of the genes considered in this study (50 total) we retrieved the corresponding upstream regions from *C. hominis*. The intergenic regions between the two species are 95% identical. As expected, we could identify the exact same motifs in all upstream regions of the corresponding orthologs in *C. hominis*, except in cases where sufficient upstream sequence was unavailable due to unfinished genome sequence (9 genes, indicated by an asterisk in Table 1).

Comparative analyses of upstream regions in *Toxoplasma gondii*, a more distant apicomplexan does not reveal the presence of the same conserved motifs in the groups studied (data not shown). This is not surprising considering the evolutionary distance between these species, and indicates that other apicomplexans may not serve as appropriate model systems for exploring the role of these *C. parvum *motifs.

## Discussion

Eukaryotic gene-regulation is a complex process that is regulated at various levels: epigenetic control via chromatin modification and reorganization; transcriptional control via proteins (transcription factors) that recognize specific signals in the DNA sequence [17]; post-transcriptional regulation at the mRNA level [18] and translational and post-translational control [19]. We chose to look for conserved *cis *elements that may be representative of transcriptional regulation in *C. parvum *as this mechanism is most tractable to a *de novo *computational approach (to identify candidate motifs in the absence of any prior knowledge about the nature or organization of regulatory regions in this system). Our study was restricted to the 5' upstream regions of each gene in consideration, and did not consider 3' regions where *cis*-regulatory signals can also, presumably, exist.

There is significant evidence for transcriptional regulation in apicomplexan parasites. Microarray analyses in the apicomplexan parasite *Plasmodium falciparum *reveal a tightly controlled cascade of gene expression as reflected by the production of specific transcripts during the various erythrocytic developmental stages [20,21]. Serial analysis of gene expression in another apicomplexan *Toxoplasma gondii *shows that unique stage-specific mRNAs are expressed during the course of its life cycle in the intermediate host [22]. Recently, *T. gondii *has also been shown to contain a rich repertoire of chromatin and histone modifying enzymes found to play a role in stage-specific gene-expression [23]. However, in both *T. gondii *and *P. falciparum*, (barring a few exceptions) co-expressed genes are not clustered within a region on a chromosome indicating that additional non-structural control mechanisms are involved in their regulation.

*Cryptosporidium parvum *is characterized by a compact genome (3952 protein coding genes in 9.1 Mb) and small intergenic regions (566 bp on an average). Genes are monocistronic and fewer than 20% of the genes are thought to contain introns [4,5], implying that gene-regulatory signals would likely be located in gene-proximal regions [24]. Previous studies of gene expression in *C. parvum *have examined genes clustered in the genome and those that are not [4,11,25]. Genomic clustering and co-expression has been observed in the *C. parvum *Large Secretory Proteins. The Cp LSP gene family exists as a cluster of seven adjacent genes on chromosome 7. These genes are co-expressed during *in vitro *development as shown by real-time PCR experiments [4]. The co-expression of these clustered genes can be a function of shared control elements duplicated during expansion of the gene family or the result of epigenetic regulation. We have provided evidence for the existence of a conserved upstream element (Figure 2b) that could possibly behave as a *cis*-acting signal to drive co-expression. Other groups of co-expressed genes are distributed throughout the genome [11,25] indicating gene-specific control of expression.

Apicomplexan parasites still present a challenge when discussing mechanisms of *cis-*regulatory transcriptional control. Experimentally dissected promoters in *T. gondii *have not revealed the presence of known canonical eukaryotic promoter elements such as the TATA box. Independent gene-specific studies have revealed the presence of non-canonical regulatory elements in upstream regions of some genes in *T. gondii *[6-8] and genome-wide studies in *P. falciparum *have indicated the presence of putative regulatory sequences correlated with expression profiles [26,27]. Preliminary genome-wide analyses of encoded proteins in various apicomplexans has revealed a reduced transcriptional machinery [4,28]. However, more than half of the predicted proteins in *C. parvum *(and other apicomplexan genomes) are hypothetical proteins. We hypothesize that the regulatory machinery in these parasites exists, but is so divergent that it cannot be identified by conventional similarity-based methods. Indeed, more sensitive, sequence-based search methods in *P. falciparum *have recently revealed the presence of basal transcriptional factors that were previously believed to be absent [26,27]. Other sensitive profile-based searches have reported the presence of a specific transcription factor ApiAP2 in *Plasmodium*, *Cryptosporidium *and *Theileria *spp. [29].

The motivation for our study was to use genomic sequence information to infer the existence of putative *cis*-regulatory motifs in *C. parvum*. Most published methods used to identify *cis*-regulatory elements build upon *a priori *knowledge of regulatory structures or expression patterns. Pattern finding algorithms are then trained, based on what is known about the organization and structure of regulons to identify additional elements. Such studies are not currently possible in *C. parvum *with traditional approaches like microarrays. We conducted a *de novo *search for conserved, over-represented short sequences in the upstream regions of genes that were grouped by metabolic function. We used two different algorithms and selected motifs predicted by both as extra evidence of significance. Both algorithms were provided with a background training set of all 3396 upstream intergenic regions in the genome to find statistically significant, over-represented motifs within the specified data sets (see methods). To determine if our findings had any functional significance, expression patterns for these genes were determined by real-time PCR experiments and the findings were correlated.

Our studies identified conserved upstream motifs that could possibly serve as recognition sites for hypothetical regulatory proteins in *C. parvum*. Biological sequences are non-random. The presence of conserved motifs in the upstream regions of genes that also demonstrate a similar expression profile indicates a possible biological function for these motifs. The actual biological role of these identified motifs remains to be determined. A possible function in splicing, post-transcriptional regulation and/or mRNA stability cannot be ruled out. Unfortunately, given the current limits of the system, experiments focused on characterizing these functions cannot be performed. However, these motifs represent an exciting starting point to investigate the presence of specific trans-acting factors in *C. parvum *that may bind these *cis*-elements. We find that specific motifs emerge from different groups of genes studied, and no common motif across all the groups could be identified. It would be hard to believe that generalized transcription factors do not exist. One limitation of our method is the lack of transcriptional start site information for genes in *C. parvum *owing to the severe paucity of EST sequences available. Aligning sequences based on transcriptional start site would be more informative with respect to revealing the presence of a possible global pattern present at a fixed location from the transcriptional start. Our study is also hindered by the AT-richness of the *C. parvum *genome (>70% in the intergenic regions). This biases the statistical significance of non A-T rich motifs as found over the background model. As more expression profiles are determined, the search can be enhanced by grouping genes based on their expression profiles into larger sets and searching for conserved patterns within.

We used two different pattern-finding tools to add to the evidence for selection of candidate motifs. MEME and AlignACE operate on different underlying algorithms and hence perform differently. In this study, the two programs displayed a fair degree of agreement in motifs reported in the top 3–5 results. However, the best motif for each program rarely corresponded to the best motif as reported by the other program, and the motifs reported from both programs were rarely 100% identical. This is because of the inherent differences between the two algorithms and the criteria they employ to identify significant motifs. We used positional information to deduce results that overlapped between the two programs and picked candidate motifs accordingly.

The motifs identified in *C. parvum *could not be found in corresponding orthologs in more distant apicomplexan species such as *Toxoplasma*, indicating that other apicomplexans may not serve as a suitable model system for *C. parvum *in this regard. Indeed, the most pressing need is to develop better experimental techniques to test bioinformatics predictions in *C. parvum *itself. Our laboratory has applied this same method in the related apicomplexan parasite *T. gondii *where well-developed molecular genetic methods exist to transform parasites and carry out reporter expression assays. These expression studies have revealed a definite function for some candidate motifs identified in this organism [Unpublished data, Mullapudi *et al*]. The study outlined here in *C. parvum *can contribute to the development of a database of "putative *cis*-regulatory elements" that will provide researchers with a starting point to investigate gene-regulation in this parasite when the experimental tools become available. This resource would help alleviate the need for traditional "promoter-bashing" approaches and speed the progress of experiments aimed at characterizing transcriptional regulatory elements.

## Conclusion

This is one of the first attempts to characterize *cis*-regulatory elements in the absence of any previously characterized elements and limited expression data. Using *de novo *pattern finding, we have identified specific DNA motifs that are conserved upstream of genes belonging to the same metabolic pathway or gene family. We have demonstrated the co-expression of these genes (often in subsets) using comparative real-time-PCR experiments thus establishing evidence for these conserved motifs as putative *cis*-regulatory elements. Given the lack of prior information concerning expression patterns and organization of promoters in *C. parvum*, the motifs identified here mark a starting point for the investigation of gene regulation in this important human pathogen.

## Methods

### Gene prediction and retrieval of intergenic regions

We used GLIMMER [30] to predict genes on the *C. parvum *genome, wrote scripts in PERL to extract gene-coordinate information and created the intergenic regions database (3396 sequences). To exclude the possibility of including coding regions in this set, a BLASTX was performed against known annotated *C. parvum *proteins using the set of intergenic regions as the query. 1000 sequences that contained portions of 100% identity to fragments of *C. parvum *protein sequences were trimmed to remove the protein coding regions. The upstream region for a gene refers to the entire intergenic region upstream of the translational start.

### Organization of genes into functional groups

In the absence of expression information, we classified genes into putatively co-regulated groups based on their function. To identify genes belonging to each pathway/group, we made use of existing annotation, and BLASTP searches using orthologues from the related apicomplexans *Plasmodium falciparum *and *Toxoplasma gondii*.

### Identification of conserved motifs in the upstream regions

We applied two pattern finding algorithms MEME and AlignACE to identify *de novo *patterns in the upstream regions. We used a background model based on the entire set of intergenic regions (3396 sequences) in *C. parvum *to train these algorithms. To identify patterns, the length range was set between 8 to 20 bp, and three different modes of occurrence were specified. The top 10 non-overlapping results from each algorithm were examined and compared, and the best motifs predicted by both algorithms were selected. We used WebLogo [31] to create sequence logos to represent the best motifs found in each search.

### *C. parvum *culture and RNA isolation

*C. Parvum *infected cultures – Human ileocecal adenocarcinoma cells (HCT-8, ATCC CCL-244; American Type Culture Collection, Rockville, MD.) were plated on 10 cm plates and cultured to approximately 70% confluency in RPMI 1640 medium supplemented with 10% fetal bovine serum (FBS), sodium pyruvate, and antibiotics/antimycotic solution (100U penicillin G/ml, 100 μg streptomycin/ml and 0.25 μg amphotericin B/ml). *C. parvum *oocysts (Iowa strain) harvested from calves were purchased commercially (Pleasant Hill farms), stored at 4°C and used for *in vitro *infections prior to three months of age as previously described [11]. Briefly, oocysts were surface sterilized by treatment with a 1:3 dilution of Clorox bleach (1 ml/3 × 107oocysts) on ice for 7 minutes, washed repeatedly with Hank's buffered saline solution (HBSS), and stored in Cp media [RPMI 1640 media containing 10% fetal bovine serum, 15 mM Hepes, 50 mM glucose, 0.1u bovine insulin/ml, 35 μg ascorbic acid/ml, 1.0 μg folic acid/ml, 4.0 μg 4-aminobenzoic acid/ml, 2.0 μg calcium pantothenate/ml, 100U of penicillin G/ml, 100 μg of streptomycin/ml and 0.25 μg of amphotericin B/ml (pH 7.4)] at 4°C overnight. HCT-8 cultures were switched to Cp media approximately 18 hours prior to infection. Oocysts were warmed to room temperature for 30 minutes, and added to HCT-8 monolayers at a 1:1 ratio. Cells were incubated in a humidified incubator at 37°C in an atmosphere containing 5% CO_2_. Following a 2 h excystation period at 37°C, cells were washed repeatedly with warm HBSS and incubated at 37°C in fresh Cp media until harvested. Infection was estimated to be between 70%-90% depending on the batch and storage period of oocysts. Total RNA was harvested in TRIzol reagent (Invitrogen) at 2, 6, 12, 24, 48, and 72 hours post infection and purified by following manufacture's instructions. Mock-infected cultures, cultures treated identically with the exception of infection, were harvested at exact time points as *C. parvum *infected cultures. Three independent time-courses were plated, infected, and harvested for this study.

### Comparative real-time PCR

To investigate gene expression during *C. parvum in vitro *development, gene-specific primers (see additional file 1 for primer sequences) were designed and used in comparative real-time PCR analysis. First strand cDNA was made using manufacturer (Invitrogen) protocols. Briefly, 2 μg of total RNA that had been previously DNased following manufacturer instructions (Turbo DNase, Ambion) was mixed with 0.5 μg of random hexamer and RNase-free water (to bring up the volume to 12 μl), heated at 70°C for 10 min, and cooled on ice. To this mixture was added a 7 μl aliquot, consisting of 4 μl of 5X first strand buffer, 2 μl of 100 mM dithiothreitol, and 1 μl of 10 mM dNTP mixture. The reaction was equilibrated at 42°C for 2 min on an iCycler (BioRad), after which 1 μl (200 U) of SuperScript II RT was applied. The reaction mixture was then incubated at 42°C for 50 min, heated at 70°C for 15 min, and held at 4°C. Identical reactions were set up without the addition of reverse transcriptase to test for the presence of contaminating genomic DNA using primers to *C. parvum *rRNA and 50 cycles of PCR under standard conditions. cDNA made from RNA containing no detectable product in the above PCR reaction was used for comparative real-time PCR.

Comparative real-time PCR was performed using a Stratagene Mx3000 *P *real-time instrument in a 96 well format. Due to the sensitivity of the machine the amount of ROX normalizing dye needed to be reduced in the reactions. Therefore, 20 μl reactions were set-up using a modified master mix consisting of 10 μl 2X SYBR master mix (1 part SYBR green master mix containing ROX dye to 5 parts SYBR green master mix without ROX dye), 2 η mol of each primer and water to 13 μl. cDNA was diluted 1:150 and 7 μl of template was added to each reaction. After an initial denaturation at 94°C for 2 min, the reaction mixture underwent 42 cycles of denaturation at 94°C for 30 sec, annealing at 58 or 59°C for 20 sec, and extension at 68°C for 30 sec. Fluorescence was read after the end of each annealing cycle. Following the end of amplification cycles, a melting curve was run. This cycle started with an initial denaturation step at 94°C followed by annealing at 56°C. Melting was performed by increasing the temperature in single degree increments until the temperature reached 94°C. Fluorescence of SYBR green was read at each increase and the data was plotted onto a graph using the Mx3000 *P *software. cDNA made from RNA harvested from three independent timecourses was run in duplicate reactions and the average Ct value of each duplicate was determined using the Stratagene Mx3000 *P *software. As the number of developing *C. parvum *life stages within infected cells changes over time, primers specific for *C. parvum *18S rRNA were used to normalize the amount of cDNA product of the target genes to that of *C. parvum *rRNA in the same sample. Due to the fact that rRNA is much more abundant than any specific mRNAs, the cDNA was diluted an additional 40 times and reactions were set up as above using three replicates of each time-course. Average *C. parvum *rRNA Ct values for each time-course was determined as above. An additional single time point of each time-course was run using *C. parvum *rRNA primers on each plate containing target genes to test the consistency of runs from plate to plate.

To determine comparative gene expression of a target gene, the average Ct values of all time points (expressed in log scale) of a single biological replicate were linearized and the ratio of expression for each time point was determined by dividing each by the product of the time point with the lowest expression (highest Ct). The ratio of expression for each time point of *C. parvum *rRNA expression was determined exactly as above. Relative target gene expression was determined by normalizing the ratio of the target gene expression to the ratio of *C. parvum *rRNA expression. Values from three biological replicates were imported into Prism (GraphPad Software). The mean and standard errors for each time point was determined using Prism's statistical package and the resulting graph was normalized to 100% maximum expression.

## Abbreviations

RT – Reverse Transcription; MEME – Multiple Em for Motif Elicitation; AlignACE – Aligns Nucleic Acid Conserved Elements

## Authors' contributions

NM and JCK designed the analysis and NM conducted the computational analyses. CAL conducted the comparative RT-PCR experiments. NM drafted the initial manuscript and JCK provided comments and critical revisions to the manuscript. JCK and MSA coordinated the study. All authors have read and approved the final manuscript.

## Supplementary Material

Additional File 2 Top ten motifs reported by each algorithm. Top scoring motifs found by both MEME and AlignACE and their respective E-values and scores. Candidate motifs reported in Figure 2 and 3 are denoted in bold. Note that the motifs found by the two programs are never identical. We used positional information to deduce overlapping motifs, and used the motifs identified by AlignACE to represent the consensus since AlignACE found more than one occurrence of the same motif within the same sequence and hence produced a more degenerate motif. Click here for file

Additional File 3 Information content and occurrences of candidate motifs reported in the paper. This table describes the Information content values as determined by MEME for each of the candidate motifs reported in the paper. Additionally, the occurrences of these motifs within their respective sub-set and the global occurrence within the whole genome (in intergenic regions) is reported. Click here for file

Additional File 1 Primer sequences for comparative PCR experiments. Oligonucleotide primers used in this study. L= left or forward primer; R= right or reverse primer. Click here for file

## Figures and Tables

**Table 1:** Genes used in this study

**GENE NAME**	**ABBREV**	**LENGTH OF UPSTREAM**	**GENE ID**
**GLYCOLYSIS**			
Hexokinase	HK	852	cgd6_3800
Phosphoglycerokinase	PGK	986	cgd7_910
Phosphofructokinase 1	PFK1	455	cgd3_1400
Phospphofructokinase 2	PFK2	575	cgd2_2130
Enolase	Eno	364	cgd5_1960
Glyceraldehyde-3-phosphate dehydrogenase	GAPDH	837	cgd6_3790
Fructose-bis-phosphate Aldolase	Ald	482	cgd1_3020
Glucose-6-phosphate isomerase	G6PI	225	cgd2_3200
Lactate Dehydrogenase	LDH	891	cgd7_480
Phosphoglucomutase	PGM	606	cgd7_4270*
Pyruvate kinase	PyK	718	cgd1_2040
Triose Phosphate Isomerase	TPI	500	cgd1_3040
**NUCLEOTIDE SALVAGE**			
Inosine monophosphate dehydrogenase	IMPDH	1065	cgd6_20*
Adenosine Transporter	AT	879	cgd2_310
Cytidine Triphosphate Synthase	CTPS	856	cgd5_1710
Guanidine Monophosphate Synthase	GMPS	423	cgd5_4520*
RibonucleosideDiphosphate Reductase	RDPR	627	cgd6_1950
deoxycytidine Monophosphate Deaminase	dCMPD	512	cgd2_2780
Dihydrofolate reductase-Thymidyl Synthase	DHFR-TS	481	cgd4_4460
Adenosine Kinase	AK	170	cgd8_2370*
Uridine Kinase	UK	404	cgd8_2810
Thymidine Kinase	TK	362	cgd5_4440
**DNA REPLICATION**			
DNA polymerase -α-subunit	DNA pol α	879	cgd8_870
DNA polymerase -δ-catalytic subunit	DNA pol δ	234	cgd6_4410*
MCM 10p-like	MCM 10p	92	cgd6_1710
MCM 2-like	MCM2	485	cgd2_1100
MCM3-associated	MCM3-a	882	cgd3_3570
MCM3-like	MCM3	370	cgd2_1600
MCM4-like	MCM4	638	cgd2_1250
MCM5-like	MCM5	155	cgd7_2920
MCM6-like	MCM6	604	cgd6_240*
MCM7-like	MCM7	374	cgd4_970
ORC/CDC6-like	CDC6	1301	cgd4_4320
RP-A ssb protein	RP-A	730	cgd2_4080
RAD24/RF-C activator	RAD24	571	cgd7_2660
MCM7 domain containing	MCM7-c	760	cgd8_3360
OOCYST WALL PROTEINS			
COWP1	COWP1	366	cgd6_2090
COWP3	COWP3	530	cgd4_670
COWP4	COWP4	197	cgd8_3350
COWP5	COWP5	170	cgd7_5150
COWP6	COWP6	688	cgd4_3090
COWP8	COWP8	604	cgd6_200
COWP9	COWP9	170	cgd6_210*
**LARGE SECRETORY PROTEINS**			
LSP1	LSP1	370	cgd7_3800*
LSP2	LSP2	370	cgd7_3810
LSP3	LSP3	225	cgd7_3820*
LSP4	LSP4	454	cgd7_3830
LSP5	LSP5	142	cgd7_3840
LSP6	LSP6	257	cgd7_3860
LSP7	LSP7	257	cgd7_3870

Descriptions of the genes used in this study, organized by functional group. The lengths of the respective upstream sequences (distance until the previous CDS) and annotated gene ids for each *C. parvum *gene are shown. Gene ids marked by a * are those for which corresponding ortholog information in *C. hominis *could not be obtained.
